# Clip‐Centered Common Bile Duct Stones Managed by Endoscopic Sphincterotomy Plus Endoscopic Papillary Large Balloon Dilation Years After Cholecystectomy

**DOI:** 10.1002/deo2.70280

**Published:** 2026-01-13

**Authors:** Nobuhiko Fukuba, Erito Ando, Taisuke Ohmachi, Yasuhide Kodama, Masaki Onoe, Kousaku Kawashima, Norihisa Ishimura, Shunji Ishihara

**Affiliations:** ^1^ Department of Internal Medicine II Shimane University Faculty of Medicine Shimane Japan

**Keywords:** clip, common bile duct stone, endoscopic papillary large balloon dilation, laparoscopic cholecystectomy, migration

## Abstract

Migration of clips used in laparoscopic cholecystectomy (LC) is rare, but when a clip migrates into the bile duct, it can serve as a nucleus for common bile duct stone formation and trigger acute cholangitis. An elderly woman in her 80s underwent LC for cholecystolithiasis at another hospital in year X. Abdominal computed tomography in year X+1 revealed that two clips had migrated into the bile duct, but this was not noted at the time, and she remained asymptomatic. In year X+5, she underwent bioprosthetic aortic valve replacement for severe aortic regurgitation. In year X+6, she presented to our emergency department with fever and right upper quadrant pain, and she was diagnosed with acute cholangitis. On the day of admission, only drainage was performed, resulting in rapid improvement of the inflammation. On the sixth hospital day, endoscopic sphincterotomy and endoscopic papillary large balloon dilation (EPLBD) were performed, and the stones were completely removed. Infrared analysis of the retrieved stones showed that over 98% consisted of calcium bilirubinate. Even if clip migration occurs relatively early after LC, there may be a time lag of several years before stone formation and symptom onset; EPLBD achieved complete, safe removal without lithotripsy.

## 1 Introduction

Since its introduction in the late 1980s [1], laparoscopic cholecystectomy (LC) has become the standard procedure for cholecystolithiasis. However, migration of metal clips used in LC into the bile duct was first reported in 1992 [2], and such migrated clips can serve as a nucleus for common bile duct stone formation, leading to acute cholangitis. This case is reported because, although clip migration into the bile duct was confirmed 1 year after LC, acute cholangitis did not develop until 6 years later, and the stones were successfully removed endoscopically.

## 2 Case Report

Patient: Female, in her 80s.

Chief complaints: Fever, right upper quadrant pain.

Past history: LC for cholecystolithiasis at another hospital in year X and aortic valve replacement surgery for aortic insufficiency in year X+5.

Medications: Aspirin.

**FIGURE 1:**
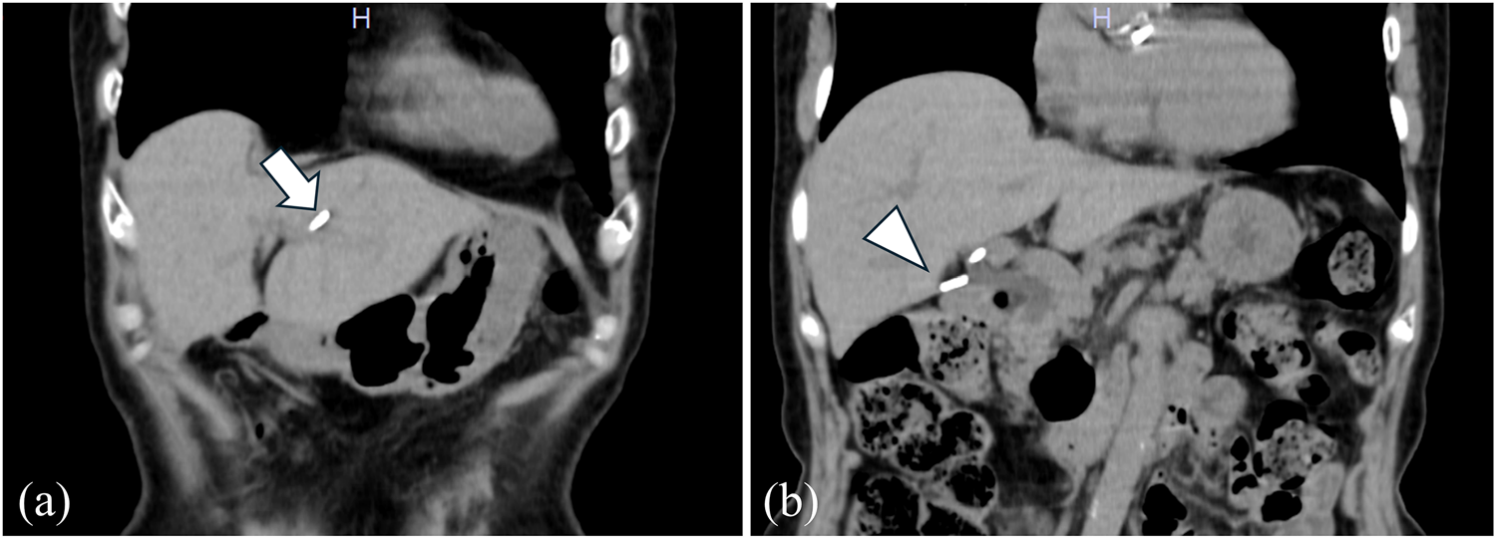
Non‐contrast abdominal computed tomography (CT) scan 1 year after cholecystectomy (coronal view). (a) One of the two clips is misplaced in the left hepatic duct, and the other is misplaced in the common hepatic duct (arrow). (b) Another clip remains in the cystic duct (arrowhead). Figures 1 (a) and (b) are both 497 × 415 mm, 330 dpi.

Clinical course:

Non‐contrast abdominal computed tomography (CT) performed in year X+1 for evaluation of another disease revealed that two clips had migrated into the bile duct (Figure 1a) and one clip remained in the cystic duct (Figure 1b), but this finding was not pointed out, and no treatment was given due to lack of symptoms. In year X+6, she developed fever and right upper quadrant pain and visited our emergency department. On arrival: Height 151 cm, weight 46 kg, temperature 38.8°C, blood pressure 80/56 mmHg, pulse 92/min, SpO_2_ 93% (room air), jaundice of the conjunctiva, tenderness in the right hypochondrium, flat and soft abdomen.

On arrival at the emergency department, laboratory tests showed cholestasis: total bilirubin 5.9 mg/dL; aspartate and alanine aminotransferases 287/274 U/L; alkaline phosphatase 916 U/L; amylase 421 IU/L; C‐reactive protein 2.25 mg/dL. The blood count showed white blood cells 18.2 × 10^3^/µL, hemoglobin 12.8 g/dL, and platelets 9.9 × 10^4^/µL.

**FIGURE 2:**
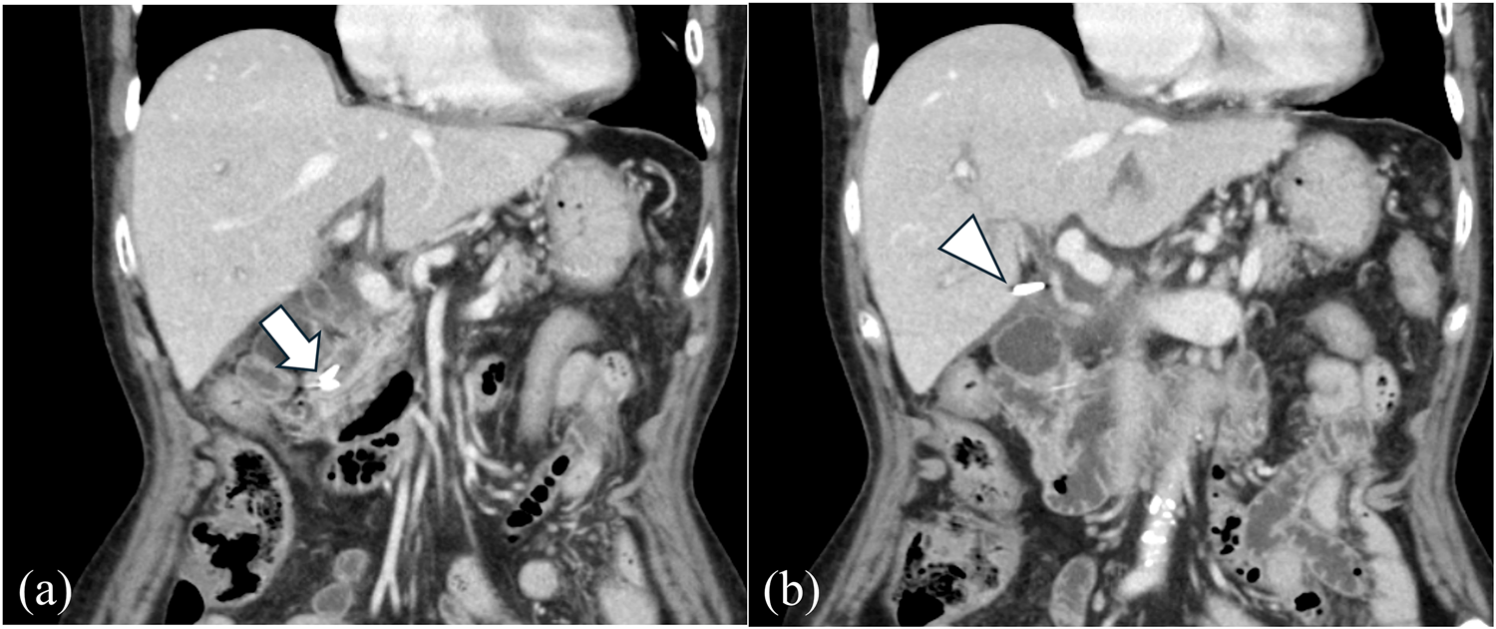
Contrast‐enhanced abdominal computed tomography (CT) scan at the time of the recent hospitalization (coronal view). (a) Two clips overlap in the lower bile duct (arrow). (b) The clip that had been closing the cystic duct on the previous CT scan was still in place (arrowhead). Figures 2 (a) and (b) are both 483 × 413 mm, 330 dpi.

A contrast‐enhanced CT scan revealed that the common bile duct was dilated to 11 mm in diameter, and two linear, high‐density structures measuring 7–8 mm in length were observed in the lower bile duct (Figure 2a), and the clip that had been closing the cystic duct on the previous CT scan was still in place (Figure 2b). These findings were consistent with two surgical clips that had already migrated into the bile duct 1 year after the cholecystectomy.

**FIGURE 3:**
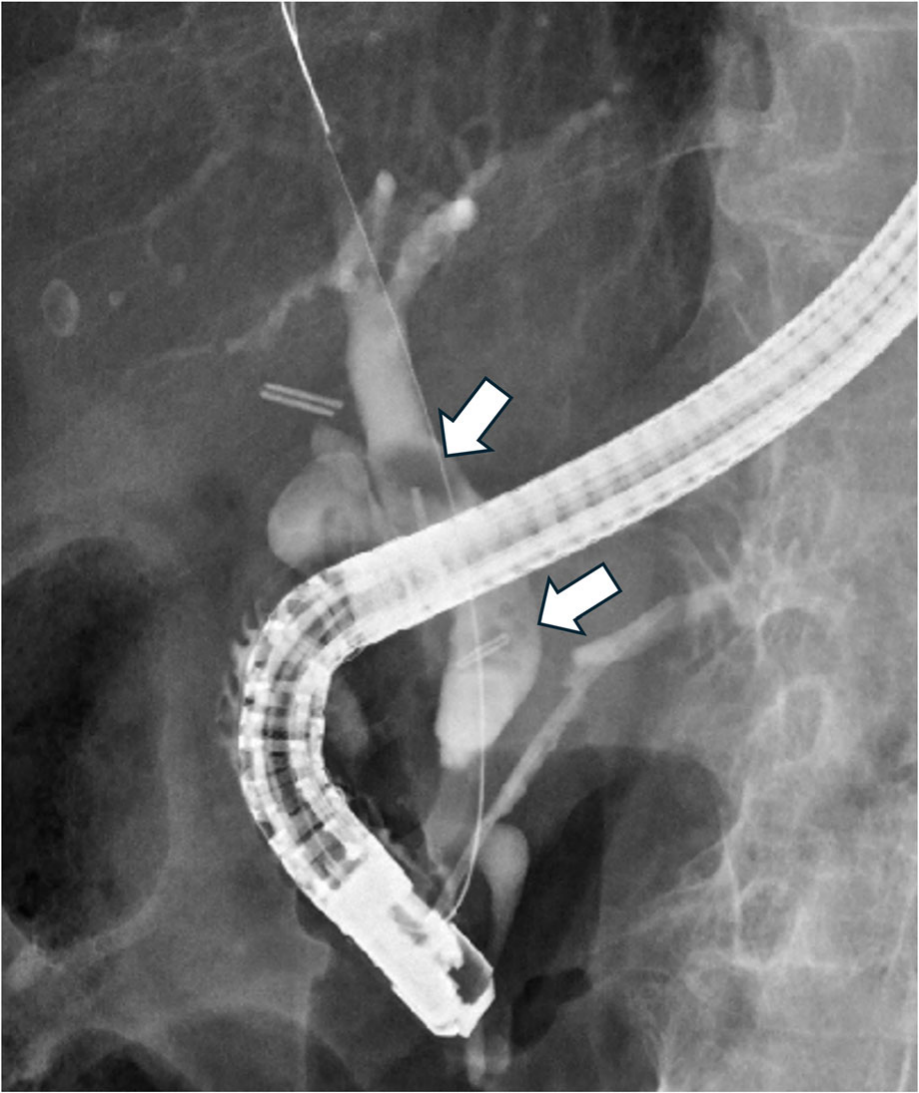
First endoscopic retrograde cholangiopancreatography (ERCP). There are two filling defects in the common bile duct, each containing a clip in the center (arrow). Figure 3 is 160 × 135 mm, 330 dpi.

**FIGURE 4:**
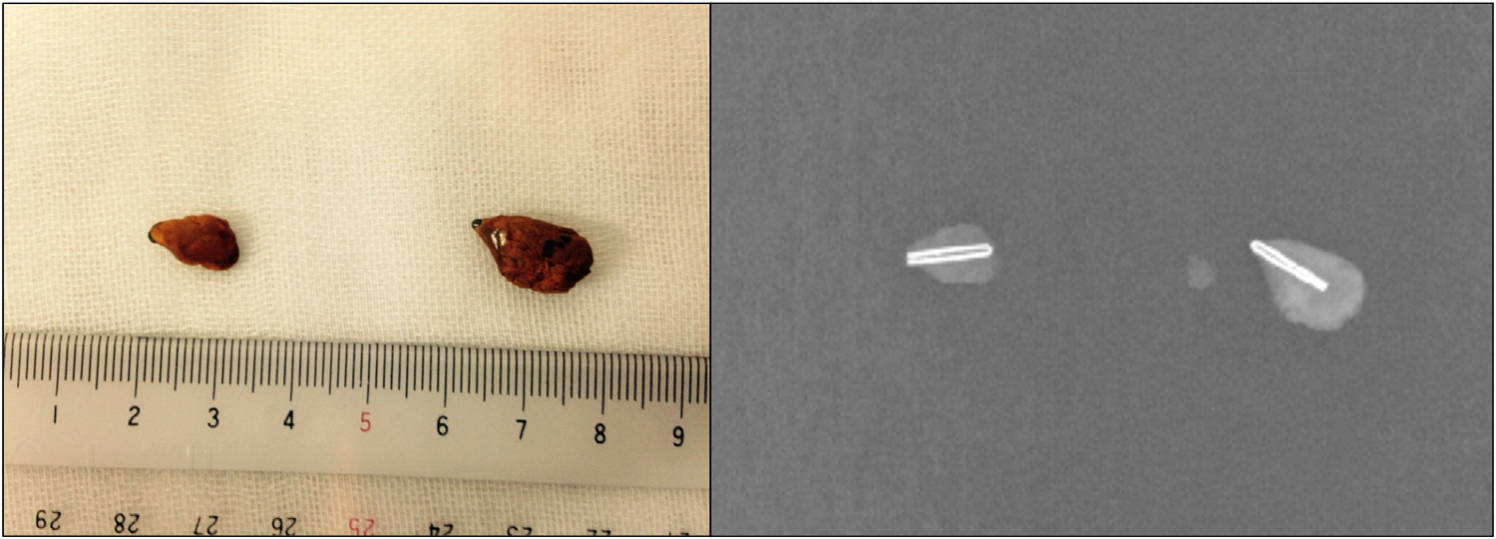
(a) A macroscopic image of the stones retrieved orally using a retrieval net. (b) A fluoroscopic image of the common bile duct stone, showing the clip encasing it.

On the day of admission, because she was on antiplatelet therapy (aspirin) after aortic valve replacement surgery, endoscopic sphincterotomy (EST) was deferred due to concerns about the risk of bleeding. Plastic stents were placed in the bile duct and pancreatic duct, leading to rapid improvement in cholangitis. On the sixth hospital day, sufficient cholangiography was performed, revealing two floating 10‐mm defects in the bile duct, each containing a clip at its center (Figure 3). Lithotripsy was one treatment option, but there was a risk that if the gallstone encasing the metal clip were to break apart, the exposed metal clip could damage the bile duct wall when it was expelled into the duodenum. To avoid this, EST and endoscopic papillary large balloon dilation (EPLBD) were performed. The maximum size of the stones was 10 mm, but the maximum diameter of the bile duct was 12 mm. In order to minimize resistance when dragging the stones from the bile duct into the duodenum, we selected the GIGA II (Century Medical, Inc., Tokyo), which can dilate up to 12 mm at low pressure, and carefully inflated it. The waist disappeared at 2 atm, so we maintained this pressure for 1 minute and then stopped dilating, allowing the stones to be smoothly expelled into the duodenum without fragmentation. The stones were then retrieved orally using a retrieval net for further analysis (Figure 4a). Figure 4b shows a fluoroscopic image of the common bile duct stone, showing the clip encasing it. Fourier‐transform infrared spectroscopy (FT‐IR) revealed that the retrieved stones consisted of more than 98% calcium bilirubinate (Figure S1).

## 3 Discussion

Clip migration into the bile duct after LC has been increasingly reported [2]. Proposed mechanisms include cystic‐duct stump necrosis from hepatic compression and bile‐duct erosion due to malposition and inflammation, allowing the clip to become a nidus for gallstones [3, 4]. Risk is higher with a short cystic‐duct stump, placement near the cystic‐duct–common bile duct junction [4], a steep clip–duct angle, or inadequate grasp requiring multiple clips. In this case, there were no images taken immediately after surgery, so the angle at which the clip intersected with the cystic duct could not be evaluated, but three clips were applied, and two of them had migrated into the bile duct. It is possible that the end of the clip that was incompletely grasping the cystic duct migrated into the cystic duct.

On the other hand, there are multiple methods for closing the cystic duct during LC, and the phenomenon of migration into the common bile duct, as seen in this case, does not appear to be limited to metal clips. Since the 2010s, there has been an increasing number of case reports of locking polymer clips, which are now widely used, also migrating into the bile duct and forming common bile duct stones with the clip as the nucleus [5, 6]. Furthermore, there have also been reports of non‐absorbable sutures migrating into the bile duct and becoming the nucleus of a gallstone (suture‐stone) [7]. In recent years, clipless cholecystectomy using energy devices has also become common, and in the future, common bile duct stones caused by the accidental insertion of foreign objects may disappear altogether.

In this case, clip migration was already present 1 year after LC, but acute cholangitis developed 5 years later, indicating a significant time lag between migration and symptom onset. Previous reports show the interval from LC to cholangitis ranges from weeks to over 10 years, with a median of 26 months [8]. In this case, the calcium bilirubinate stones identified by infrared analysis are the most common type of common bile duct stones, and they are formed by the precipitation of calcium bilirubinate due to infection or bile stasis. This case revealed that the migration of the clip into the bile duct occurred in the early postoperative period, but it also became clear that symptoms do not necessarily appear immediately. The fact that asymptomatic clip migration can occur several years before the onset of symptomatic biliary events provides important insight into the prediction of late complications after cholecystectomy. However, it is unclear whether the gallstones formed slowly after the migration or whether the heart valve surgery performed 5 years after the cholecystectomy accelerated the formation of the gallstones.

Treatment of common bile duct stones caused by clip migration into the bile duct can mostly be managed with endoscopic retrograde cholangiopancreatography‐related procedures, similar to typical common bile duct stones [9]. In this case, the patient was taking antiplatelet therapy (aspirin), and in accordance with the Tokyo Guidelines 2018, drainage was prioritized, and later we safely and completely removed the stone using EST and EPLBD. EPLBD is an effective endoscopic treatment method for common bile duct stones that allows for the removal of relatively large bile duct stones, 10 mm or more in size, and especially those 15 mm or more, without needing to fragment them. In this case, the combined use of EST and EPLBD enabled the safe removal of a sharp metal clip wrapped in bilirubin calcium. Previous reports have often shown that endoscopic removal is performed for bile duct stones caused by migrated clips, but there have also been reported cases in which stones were safely removed by surgical resection [10]. We summarized additional published cases descriptively in TableS1. EST and EPLBD are extremely useful from a medical safety perspective in the treatment of bile duct stones caused by migrated clips.

Clip migration after LC can occur relatively early, but there may be a long latent period before symptomatic common bile duct stone formation and acute cholangitis. Long‐term imaging and endoscopic follow‐up should be considered in cases where clip migration is suspected.

## Author Contributions

**Nobuhiko Fukuba**: study conception and design; clinical management; data collection; drafting of the manuscript; figure preparation. **Erito Ando** and **Taisuke Ohmachi**: clinical data acquisition and curation; imaging review; critical revision for clinical accuracy. **Yasuhide Kodama** and **Masaki Onoe**: imaging analysis (MRI/CT/ultrasound); figure supervision; critical revision of the imaging section. **Kousaku Kawashima**: endoscopic assessment and illustration; description of procedures; literature review. **Norihisa Ishimura**: methodological advice; interpretation of findings; critical manuscript revision. **Shunji Ishihara**: overall supervision; critical revision for important intellectual content; final approval of the version to be published.

## Conflicts of Interest

Norihisa Ishimura is an Associate Editor of DEN open and has not been involved in the peer review or editorial decisions for this manuscript.

## Funding

N/A

## Supporting information




**FIGURE S1** (a) Macroscopic image of a cross‐section of a gallstone and (b) Fourier transform infrared spectroscopy (FT‐IR) spectrum of the stone. The vertical axis represents Transmittance (%), and the horizontal axis represents Wavenumber (cm^−1^). FT‐IR showed the characteristic calcium bilirubinate triplet at ∼1666/1624/1566 cm^−^^1^ (white arrow) with supporting bands at ∼1400 and ∼1250 cm^−^^1^ (gray arrow) and a pyrrolic N–H band at ∼3398 cm^−^^1^ (black arrow).

**TABLE S1** Post‐cholecystectomy clip migration with common bile duct stones: cases, closure materials, and endoscopic/surgical management.
We summarized additional published cases descriptively; due to the journal's reference limit, only representative reports [6, 9, 10] are cited in the text.
