# Supplementary figures and images for: Clip‐Centered Common Bile Duct Stones Managed by Endoscopic Sphincterotomy Plus Endoscopic Papillary Large Balloon Dilation Years After Cholecystectomy
Source: DEN Open. 2026 Jan 13;6(1):e70280. doi: 10.1002/deo2.70280 (PMC12796956; doi:10.1002/deo2.70280)

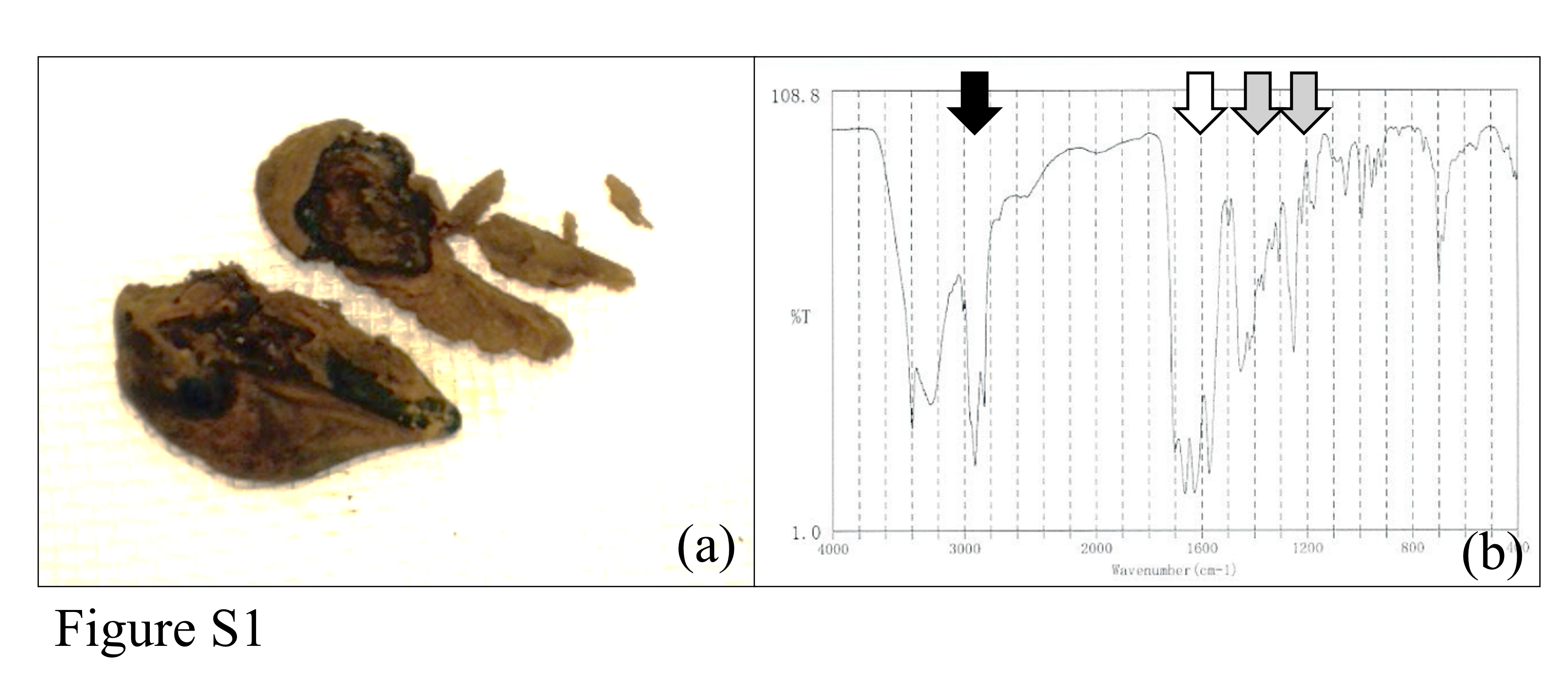

Supplement: Supplementary file 1 — FIGURE S1 (a) Macroscopic image of a cross‐section of a gallstone and (b) Fourier transform infrared spectroscopy (FT‐IR) spectrum of the stone. The vertical axis represents Transmittance (%), and the horizontal axis represents Wavenumber (cm−1). FT‐IR showed the characteristic calcium bilirubinate triplet at ∼1666/1624/1566 cm− 1 (white arrow) with supporting bands at ∼1400 and ∼1250 cm− 1 (gray arrow) and a pyrrolic N–H band at ∼3398 cm− 1 (black arrow). [file DEO2-6-e70280-s002.tif]
