# Supplementary material for: Clip‐Centered Common Bile Duct Stones Managed by Endoscopic Sphincterotomy Plus Endoscopic Papillary Large Balloon Dilation Years After Cholecystectomy
Source: DEN Open. 2026 Jan 13;6(1):e70280. doi: 10.1002/deo2.70280 (PMC12796956; doi:10.1002/deo2.70280)
Supplement: Supplementary file 2 — TABLE S1 Post‐cholecystectomy clip migration with common bile duct stones: cases, closure materials, and endoscopic/surgical management. We summarized additional published cases descriptively; due to the journal's reference limit, only representative reports [6, 9, 10] are cited in the text. [file DEO2-6-e70280-s001.docx]

Supplementary Table

| Year | First author (reference) | No. of cases | Age | Cystic duct closure material (type/material) | Treatment for CBD stone |
| --- | --- | --- | --- | --- | --- |
| 1992 | Dhalla (N/A) | 1 | NR | Ligaclip (metallic clip) | Endoscopic removal |
| 1995 | Martinez (N/A) | 2 | NR | Cystic duct stump clips (metallic clips) | Endoscopic sphincterotomy |
| 2002 | Tsumura (10) | 1 | 57 | Endo-clips (metallic clips) | Surgical removal of impacted basket catheter |
| 2003 | Hai (N/A) | 1 | 57 | Surgical clip(s) (metallic clips) | Endoscopic removal |
| 2003 | Dell’Abate (N/A) | 1 | 67 | Surgical clip (metallic clip) | Endoscopic sphincterotomy |
| 2004 | Chong (6) | 3 | 73 / 70 / 46 | Hem-o-clips (metallic clips) | Case 1: s Surgical removal; Cases 2 and 3: Endoscopic sphincterotomy; |
| 2018 | Hussameddin (N/A) | 1 | NR | Surgical clip (metallic clip) | Endoscopic sphincterotomy |
| 2019 | Kou (N/A) | 1 | 84 | Hem-o-lok clip (polymer/non-absorbable) | Laparoscopic exploration |
| 2024 | Esekiel (9) | 1 | 64 | Metallic surgical clip (metal) | ERCP with cholangioscopy-assisted removal of clip/stone complex |
